# Goal Priming in Dieters: Recent Insights and Applications

**DOI:** 10.1007/s13679-012-0009-8

**Published:** 2012-02-28

**Authors:** Esther K. Papies

**Affiliations:** 1 Department of Psychology, Utrecht University, Utrecht, The Netherlands; 2 Department of Social Psychology, Utrecht University, Postbus 80140, 3508TC Utrecht, The Netherlands

**Keywords:** Chronic dieting, Eating behavior, Overweight, Self-regulation, Toxic environment, Goal priming, Hedonic, Implementation intentions, Social psychology, Social cognition, Field experiments, Interventions

## Abstract

What are the psychological mechanisms that make dieting so challenging in our food-rich living environment? Social psychological research on goal priming provides a useful framework for answering this question, as well as implications for how to enhance dieting success. This review presents and discusses recent research which shows that attractive food cues prime the hedonic eating goal in dieters, and thus facilitate overeating. However, external cues priming the goal of weight control can be used to offset these effects and thus to facilitate dieting success, as is demonstrated in both field and laboratory experiments. In addition, recent strategies to prevent hedonic effects of attractive food, such as mindful attention, can facilitate self-regulation. These recent advances in our understanding of dieting behavior have theoretical and practical implications for how successful dieting can be facilitated, both by means of individual strategies, as well as by environmental changes.

## Introduction

Overweight and obesity are an increasingly serious and costly health issue in most Western societies. In 2007 to 2008, more than two thirds of US adults were estimated to be overweight or obese [1]. Based on the developments over the last decades in the United States, it has been suggested that by the year 2030, more than 86% of adults may be overweight or obese, more than 50% may be obese, and that by 2048, all Americans will be overweight or obese [2].

Many people try to reduce or at least control their weight by dieting [3]. However, even when successful in reducing their weight in the short term, most dieters do not succeed at maintaining their lowered body weights over an extended period of time [4], and some even regain more than before they started dieting [5, 6]. These difficulties can motivate repeated attempts at dieting to lose weight or avoid weight gain [7, 8], and can eventually turn dieters into chronic dieters (i.e., restrained eaters [9]). These chronic dieters are highly motivated to control their weight, but are typically not very successful and often experience lapses of restraint as well as weight fluctuations [10]. The psychological processes underlying their behavior are the focus of this review.

Why is it so difficult to pursue and maintain healthy weight-control behaviors? It has been argued that the current, food-rich living environments of Western societies play a crucial role in these difficulties in weight regulation [11–13]. These so-called “toxic environments” confront us with attractive, easily available food temptations with high-fat content and calories, reminding us of the pleasures of eating tasty food rather than the benefits of controlling our eating [11, 12]. Chronic dieters react particularly strongly to hedonic food cues: compared to normal eaters (i.e., those who are not chronically trying to diet), chronic dieters experience more cravings when they are exposed to attractive food, they react with increased salivation to such cues, and they are more likely to overeat in response to the smell, sight, or thoughts of attractive food, despite their explicit intentions to diet [14–18•].

## A Goal Conflict Model of Eating Behavior

Which psychological mechanisms underlie these effects of attractive food cues on chronic dieters, and how can they be counteracted? Here, I will present and discuss research suggesting that nonconscious goal priming plays a crucial role in both processes (see also [19, 20]). Based on the goal conflict model of eating behavior [12, 21–23•], I will discuss how attractive food cues activate in chronic dieters the hedonic goal of enjoying good food. In line with recent insights into the nonconscious regulation of goal-directed behavior, this leads to the inhibition of the competing goal of weight control [23•, 24]. As a result, chronic dieters are less likely to act on their weight control goal compared to their hedonic goal of enjoying good food, which could explain their cravings and overeating when exposed to attractive food cues [15, 16, 18•]. However, this approach also implies that when the goal of weight control is activated in chronic dieters (e.g. by relevant primes in the environment), this goal will be available to influence behavior, making healthier choices more likely. An increasing number of studies now demonstrate these processes of goal priming in dieters, which has important implications for facilitating successful self-regulation.

## Toward Dieting Failures: Priming the Hedonic Eating Goal

A number of recent studies on dieters’ nonconscious cognitive processes have confirmed that chronic dieters display stronger hedonic reactions to attractive food cues than normal eaters [25, 26], often without being aware of this. For example, one set of studies showed that reading behavior descriptions involving attractive food (e.g., “Bill is having a big piece of apple pie”) spontaneously activated hedonic thoughts about food (e.g., “tasty,” “delicious”) in chronic dieters, but not in normal eaters [26]. These hedonic thoughts may be interpreted as a reflection of the mental representation of a hedonic eating goal being activated by food cues. In addition, dieters’ spontaneous hedonic responses to tasty food items seem to underlie their conscious experiences of cravings for high-fat, palatable food [25]. Further support comes from recent neuroimaging studies, which have shown that perceiving palatable food triggers neural responses in gustatory and reward-related regions in the brain, and that these neural responses are especially pronounced in obese individuals and chronic dieters [27, 28]. Together, these findings suggest that the exposure to attractive food cues triggers the hedonic goal of enjoying good food in chronic dieters.

The exposure to food cues has not only been shown to trigger strong hedonic reactions, but also to guide dieters’ visual attention toward attractive food. More specifically, when chronic dieters were exposed to attractive food words, they later displayed increased attention toward tasty food items as a function of their subjective liking of this food [29]. Similar effects of the pre-exposure to attractive food on chronic dieters’ processing of food cues have been reported by van Koningsbruggen et al. [30•]. They found that chronic dieters perceive attractive food items as bigger when they have been pre-exposed to tasty food. Again, the pre-exposure to attractive food items seems to active the hedonic eating goal, which then affects the processing of goal-relevant stimuli such that more visual attention is attributed toward them and the chances of approaching and consuming the food are enhanced.

Once chronic dieters are thus focused on the hedonic aspects of the palatable food they perceive, thoughts about dieting and weight control are less likely to come to mind. Most foods that are perceived as highly attractive are also high in calories and their consumption is incompatible with the pursuit of the weight control goal. Chronic dieters are very much aware of this, and are therefore highly ambivalent toward palatable, high-calorie food [23•, 31, 32]. In other words, the hedonic goal of enjoying good food is in conflict with the goal of weight control, and the goal conflict perspective on dieting predicts that when one of these goals is activated (e.g., by an environmental goal prime), the other goal will be inhibited to prevent interference in goal pursuit [23•, 24]. In line with this perspective, subliminal priming studies show that priming the hedonic eating goal by very briefly presenting participants with attractive food words (e.g., pizza, chocolate, tasty) decreases the cognitive accessibility of the conflicting weight control goal, as measured in a lexical decision task [23•, 33]. In other words, hedonic food cues lead to the inhibition of the weight control goal, making it less likely that this long-term health goal will guide behavior in critical situations.

To sum up, attractive food primes the hedonic eating goal in chronic dieters, which prepares dieters to pursue this goal and leads to the inhibition of the conflicting goal of weight control, making unhealthy choices and overeating more likely. This may explain why the mere exposure to the smell or sight of attractive food triggers unhealthy choices and overeating in chronic dieters, despite their intentions to control their body weight by dieting [15–17].

## Preparing for Dieting Success: Priming the Weight Control Goal

Applying this goal conflict perspective to understanding dieting behavior, however, also suggests ways for improving the chances for dieting success. As the activation of the hedonic eating goal by attractive food cues leads to the inhibition of the dieting goal and thus to dieting failures, activating the dieting goal by means of dieting cues should activate the dieting goal and thus re-instate its influence on subsequent cognition and behavior. In other words, priming the weight control goal in tempting situations should help chronic dieters to focus on this goal, and thus to be more successful dieters.

In line with this reasoning, research on the cognitive effects of diet goal primes indicates that subtly reminding chronic dieters of their goal of weight control reduces their attention for the hedonic aspects of food in their environments. In a study on visual attention, Papies et al. [29] first exposed participants to attractive food stimuli to activate the hedonic eating goal, and then subliminally primed half of participants with diet-related words while their visual attention for tasty food words was measured. Results showed that the diet primes significantly reduced chronic dieters’ attentional bias toward attractive food words, compared to the no-prime condition. Similarly, in a more recent study, van Koningsbruggen et al. [30•] primed chronic dieters with their weight control goal by exposing them to a magazine cover with diet-relevant information. Then, participants were asked to estimate the size of a food item that is instrumental for pursuing the weight control goal (i.e., an apple). Results showed that participants in the diet prime condition estimated the apple as bigger, compared to the control condition in which participants were exposed to the cover of a gardening magazine. These findings suggest that diet goal primes prepare chronic dieters to act on healthy, rather than tasty, food cues in their environment. However, do such primes also work to impact actual eating behavior?

## Priming Weight Control to Regulate Eating Behavior

Research on goal priming in the domain of social psychology has provided ample evidence that subtle cues activating the mental representation of a certain goal can lead to motivated behavior in pursuit of that goal. In their seminal study on this topic, Bargh et al. [34] subtly exposed participants to words related to performing well (e.g., master, attain, achieve), and then provided them with the opportunity to exert effort on a performance task. Participants who had been primed with the goal of performing well worked harder on this task than non-primed participants, without being consciously aware of this. These findings were later replicated in various other behavioral domains, such as helping, stereotyping, drinking, socializing, seeking casual sex, or earning money (see [35] for an overview). Crucially, goal priming effects occur only when the goal that is activated by the priming cues is seen as desirable by participants (i.e., when it is indeed a goal that they want to pursue) [36–39]. Applying this principle to the domain of dieting implies that primes of the weight control goal in one’s environment should stimulate the pursuit of diet-congruent behaviors, such as eating less or making healthier choices, but only in those individuals for whom dieting is indeed a personally relevant goal.

A recent field study has provided a first test of this hypothesis. In a real-life setting, participants were exposed to food temptations and also primed with the goal of weight control, while their eating behavior was observed [18•]. The study was conducted in a local butcher’s shop, in which the smell of grilled chicken emanated from a large grill oven while customers were being served. At the same time, customers could freely sample from bite-size meat snacks on a tray placed on the counter. In the diet prime condition, a poster announced the availability of a low-calorie recipe available in the store. In the control condition, this diet poster was not present. As the dependent variable, the number of meat snacks eaten by participants was assessed unobtrusively, and to determine whether they were chronic dieters or normal eaters, they later filled in a brief questionnaire with questions on their dieting motivation.

**Fig. 1:**
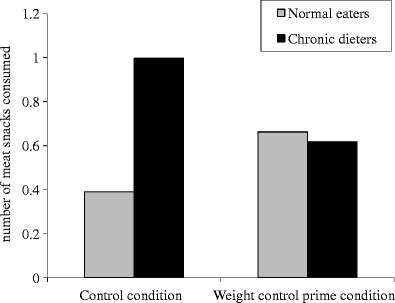
Mean number of meat snacks consumed by chronic dieters and normal eaters in the control condition, with the smell of grilled chicken present in the store, and the diet prime condition, where customers were reminded of their weight control goal by means of a poster before entering the store. (Reprinted with permission of the APA from: Papies EK, Hamstra P. Goal priming and eating behavior: Enhancing self-regulation by environmental cues. Health Psychology. 2010;29(4):384–388) [18•]

In line with earlier research on the eating behavior of chronic dieters in tempting situations [15, 16], results showed that chronic dieters ate more snacks than normal eaters in the control condition, when they were tempted by the smell of grilled chicken and the availability of free, tasty meat snacks. However, the poster with the recipe reminder reduced the number of snacks eaten by chronic dieters, while it did not affect the behavior of normal eaters (Fig. 1). Thus, the weight control prime indeed served as a goal prime, as it reduced the amount eaten only among those participants for whom dieting was a goal they wanted to pursue [18•, 35]. These findings are in line with studies by Anschutz et al. [40], who demonstrated that watching diet-relevant advertising in a laboratory setting reduced the snack consumption of chronic dieters (see also Fishbach et al. [41], Study 5).

More recently, this test of goal priming to examine whether goal primes can also lead to healthier behavior was extended to a different real-life situation, namely when eating out in a restaurant. This seems a particularly relevant setting, since the consumption of out-of-home meals has been increasing over the last years [42], while consumers underestimate the levels of fat and calories of food items offered in restaurants [43] and often overeat [42, 44, 45]. However, it may be especially difficult in a restaurant setting to remind people of their health goals and facilitate healthy behavior: customers go to a restaurant with the explicit goal of enjoying food, and this goal is continuously primed by cues such as the eating behavior of other people [26], the sight and smell of food [15, 16, 18•], and the menu itself [46]. This makes eating out a particularly relevant setting to apply goal priming for enhanced self-regulation.

Papies and Veling [47] conducted a field experiment in a café-style restaurant, where they slightly modified the menu of the restaurant to prime customers with the goal of weight control, simply by adding diet-related words to part of the menu for half of the participants. Customers’ choices were then recorded unobtrusively, and compared to choices in the no-prime control condition. In line with hypotheses and earlier findings, results showed that the diet prime led to more low-calorie, healthy dinner choices, but only among chronic dieters, and similarly, among overweight individuals. Thus, goal priming can also be applied to facilitate self-regulation in the hedonic setting of an evening out in a restaurant.

These experiments suggest that very subtle goal primes, such as words on posters or menus, magazine covers, or even subliminally presented words, can gear dieters toward pursuing their long-term health goal, even in tempting environments. Thus, due to their unobtrusive and cost-effective nature, such goal primes may constitute a useful and effective addition to the toolkit for theory-based interventions in health behavior (for a discussion, see Papies and Stroebe [19]).

## Planning for Individual Dieting Success

The research reviewed thus far has demonstrated how environmental cues priming the hedonic eating goal can facilitate hedonic eating behavior, and how cues priming the goal of weight control can facilitate the pursuit of the goal of weight control. This provides us with theoretical insight into the mechanisms of dieting failures, and with inspiration about how to modify features of the environment to help dieters be more successful. However, in our “toxic” living environment, which frequently confronts us with attractive food cues as reminders of our hedonic goals, rather than our long-term health goals, these processes are much more likely to instigate failures of self-regulation. Reminders of the dieting goal cannot easily be implemented in every part of one’s living environment. Therefore, to enable chronic dieters to successfully pursue their goal of weight control even in tempting environments without diet reminders, van Koningsbruggen et al. [48•] developed a novel technique for dieters to prime themselves with the goal of dieting in critical situations.

This goal-priming technique relies on recent advances in research on behavior regulation and on the benefits of planning one’s behavior. Specifically, forming if-then plans by means of so-called implementation intentions [49] has been shown to create a strong association between a situation in which one plans to perform a certain behavior and the behavior representation itself, so that the planned behavior is triggered automatically when the anticipated situation occurs. Implementation intentions have been shown to be a simple but powerful tool for changing behavior in a variety of domains, including health behavior (for a review and meta-analysis, see [50]).

The most common applications of implementation intentions identify a specific behavior to be performed in the anticipated situation. However, adapting this technique to enable goal priming among chronic dieters, van Koningsbruggen et al. [48•] slightly modified the use of implementation intentions, to create an association of a tempting situation with the goal of dieting, rather than a single behavior. Thus, chronic dieters planned to think of dieting when they were tempted to eat each of five different attractive food items (i.e., to plan “When I am tempted to eat chocolate, I will think of my dieting goal”), and their behavior in these situations was assessed afterwards. Results confirmed that goal priming by means of implementation intentions reduced how much of the tempting food items otherwise unsuccessful chronic dieters ate over the course of a 2-week period, thus increasing their actual success in dieting behavior [48•]. Comparable results were obtained in a similar study by Kroese et al. [51]. Thus, implementation intentions can be used as a method of individual goal priming for chronic dieters to prime themselves with the long-term goal of dieting in a critical eating situation.

## Successful Dieting

These studies clearly revealed that goal priming by implementation intentions was beneficial only for otherwise unsuccessful dieters, while it did not influence the eating behavior of those dieters who indicate that they are rather successful in regulating their own weight control behaviors [48•, 51]. What distinguishes these two groups of chronic dieters? Correlational studies suggest that successful weight loss is associated with such behaviors as consistently limiting energy intake, eating more fruit and vegetables, and not eating sweets [4, 52]. However, what are the psychological mechanisms enabling the consistent pursuit of such behaviors in some, but not other, dieters?

Several studies now suggest that the answer to this question may lie in dieters’ differential associations with attractive food cues. Specifically, an initial study on this issue examined the cognitive effects of attractive food primes [33] and revealed that for unsuccessful chronic dieters, attractive food primes lead to the inhibition of the dieting goal (see [23•]), while for successful dieters, the food primes lead to the activation of the goal of dieting (see also [41, 48•]). Thus, when confronted with tasty food, unsuccessful dieters seem to “forget” about dieting, while successful dieters are indeed reminded of their goal. Thus taking tempting food cues as reminders of one’s long-term health goal makes it much more likely that one will be able to resist the food temptations in favor of these good intentions. Successful dieters were found to be much more likely to translate their dieting intentions into behavior over a 1-week period, and, most likely as a result of their successful self-regulation, to have a lower body weight than unsuccessful participants [33].

Similarly, a later study revealed that the pattern of goal activation displayed by successful and unsuccessful dieters parallels the behavior of normal weight and overweight dieters. Here, chronic dieters with different levels of body mass index (BMI) were unobtrusively primed with attractive or neutral food cues, before their motivation to obtain tasty food was examined [53]. Results revealed that hedonic food primes differentially affected chronic dieters as a function of their level of BMI. For relatively lean dieters, attractive food primes lead to healthier choices, while for relatively heavy chronic dieters, the food primes lead to less healthy choices, compared to a neutral prime control condition. These findings suggest that attractive food cues prime the hedonic goal of eating good food in unsuccessful and heavier dieters, while they activate the dieting goal in successful and leaner chronic dieters.

An important question that still needs to be fully resolved is how these differential associations develop. How do some dieters learn to associate tasty food with watching their weight, while others associate it with indulgence? Based on social psychological research on the cognitive mechanisms underlying nonconscious goal pursuit, it has been suggested that these differential associations may develop by repeatedly pursuing either goal when confronted with tasty food cues [33, 41, 54]. Through the frequent coactivation of a situational food cue and either the weight control goal or the hedonic eating goal, these become strongly associated in mind, making the later pursuit of that same goal much more likely when in a similar situation. However, this mechanism has not yet been examined in a controlled prospective study among dieters.

Importantly, research has shown that planning to think of one’s weight control goal helped unsuccessful dieters to activate this goal in response to tempting food cues, and thus to resist food temptations in their daily lives, essentially turning them into successful dieters. Thus, this planning technique can serve to overrule unhealthy associations that may have developed by repeated unhealthy behaviors and that would otherwise lead to maintained failures of self-regulation. However, more research is needed to gain insight into how the psychological mechanism of success spontaneously develops in some dieters, enabling them to remain slim in our current “toxic” living environment.

## Strategies to Prevent Hedonic Goal Priming

In addition to goal priming strategies, recent attempts to facilitate dieting success have also included strategies to reduce the potential of palatable, high-fat food items to activate the hedonic eating goal in the first place. From the perspective of grounded cognition [55, 56], hedonic reactions toward food develop when participants spontaneously simulate the experience of eating the food they are exposed to, as well as the accompanying pleasure and reward [57•]. These hedonic eating simulations then trigger the motivation to approach and eat the food. To reduce the effects of these simulations towards food, Papies et al. [57•] developed a brief training procedure based on the principles of mindfulness [58]. Specifically, participants were trained to observe their hedonic simulations and impulsive reactions to food items in a nonjudgmental way and consider them as passing mental states, which arise and dissipate in reaction to perceiving external stimuli [58]. A series of studies using this procedure demonstrated that even a very brief training in this perspective of mindful attention was effective to eliminate participants’ automatic approach reactions to attractive food. Thus, hedonic impulses toward food can be reduced by mindful attention, which therefore offers great potential to facilitate self-regulation in the face of high-fat food, and potentially other attractive stimuli. Other training procedures to reduce impulsive approach reactions to attractive food have also been advanced recently (e.g., training participants repeatedly to withhold their responses to food temptations), and these approaches continue to be developed [59•, 60, 61].

## Conclusions

The studies reviewed here show that the exposure to attractive food cues can trigger a hedonic eating goal in chronic dieters and lead to the inhibition of the weight control goal, facilitating self-regulatory failures. However, chronic dieters can benefit from mechanisms that activate the weight control goal in critical situations, such as diet goal primes provided by the environment or by individual plans, leading to healthier behavior. Thus, the goal priming perspective on chronic dieting provides a useful framework to understand dieting failures, as well as insights into how to use goal priming methods to facilitate more successful self-regulation with regard to eating behavior, which is highly relevant given our food-rich living environment and the current “obesity epidemic” [13].

These findings can inform the development of strategies to facilitate chronic dieters’ health efforts (e.g., by thinking ahead and using implementation intentions to think of their dieting goal in tempting situations), or by devising personalized diet reminders for strategic places (e.g., the refrigerator, the cookie cupboard), to influence one’s behavior when one is about to indulge in high-fat snacks (see also [19]). Keeping tempting snacks out of sight to prevent activation of the hedonic eating goal in the first place is likely to help [62]. In addition, applying techniques to reduce the appeal of palatable, high-fat food stimuli (e.g., mindful attention [57•]) will make it easier to resist such stimuli even without relying on the activation of the dieting goal. However, given the current obesity epidemic, we may ultimately have to turn to environmental changes, too, to curb the abundance of high-calorie food in our living environment, and to make healthy alternatives available to all members of society, independent of their socioeconomic status and the neighborhood they live in [63–66]. In addition to developing effective individual strategies for behavior change, such policy changes may be necessary to facilitate the successful regulation of eating behavior and thus to prevent further large-scale weight gain [11, 19, 65, 67].
